# A retrospective study of oral tofacitinib therapy for alopecia areata^☆^

**DOI:** 10.1016/j.abd.2022.05.002

**Published:** 2023-01-20

**Authors:** Xinaida Taligare Vasconcelos Lima, Melissa Bambery, Maria Beatrice Alora

**Affiliations:** a Clinical Unit for Research Trials in Skin, Massachusetts General Hospital, Boston, Massachusetts, United States; b Dermatology Division, Internal Medicine Department, Universidade Federal do Ceará, Fortaleza, CE, Brazil

Dear Editor,

Alopecia Areata (AA) is an autoimmune non-scarring alopecia with an overall prevalence of around 2%.^1^ Treatment of moderate to severe AA is challenging. Retrospective studies have demonstrated that tofacitinib may induce hair regrowth in patients with moderate to severe AA.^2^, ^3^, ^4^, ^5^, ^6^, ^7^ While the safety and efficacy of tofacitinib in severe AA were supported in these studies, the durability of treatment has not been extensively documented.

In this retrospective cohort study, approved by Mass General Brigham Institutional Review Board, the authors have systematically explored the long-term efficacy, safety, and durability of tofacitinib for the treatment of AA. The authors were also interested in evaluating the impact of the temporary discontinuation of tofacitinib on the course of AA during the COVID pandemic.

Using a centralized clinical data registry from various hospital systems, the authors identified patients with AA that had received tofacitinib until February of 2021. Eligible patients had a diagnosis of AA and received systemic tofacitinib for a minimum of three months (Fig. 1).

**Figure 1:**
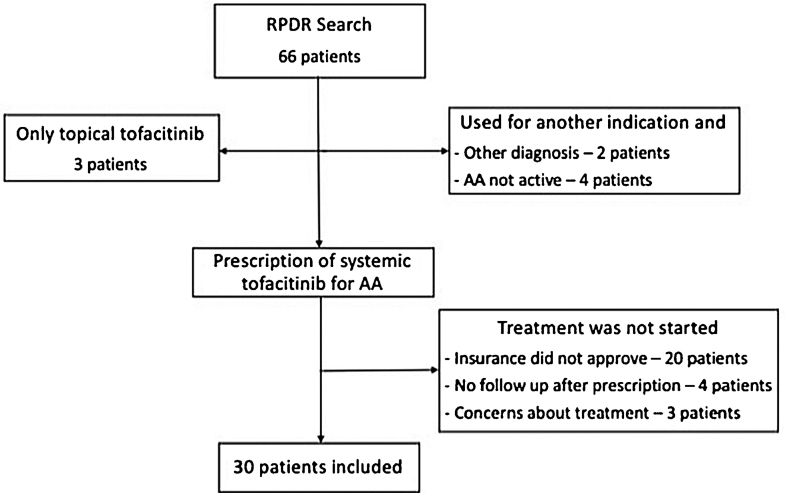
Patient selection flowchart.

Diagnosis of AA was confirmed by a review of the patient’s medical record. All patients had AA for at least six months and were followed by a dermatologist. Information on the severity or proportion of scalp involvement, when available, was collected from dermatologist notes. Most of the patients excluded never started tofacitinib (lack coverage or safety concerns). Others had not been seen after tofacitinib had been prescribed, were taking tofacitinib for another indication, and did not have concomitant AA.

Treatment response was categorized as complete (almost or total regrowth) and non-complete (partial or minimum), based on the providers’ notes. The authors evaluated demographics and disease characteristics between complete and non-complete using Mann-Whitney and Fisher Exact (SPSS 20.2, IBM, Armonk, NY).

The present study included 30 AA patients. Sixty percent had totalis or universalis and 20%, ophiasis (Table 1). AA in four patients was potentially triggered by an anti-TNF agent, used to treat an additional inflammatory disease. All patients had previously received topical or intralesional steroids and 21 had taken other systemic therapies.

**Table 1:** Patient and disease characteristics that may affect complete response.

	All patients (n = 30)	Complete Responders (n = 20)	Non-complete responders (n = 10)	p-value
**Age**^a^**, yr**	34 (12‒63)	31 (14‒64)	54 (17‒64)	0.25
**Female**	23 (76.7)	16 (80.0)	7 (70.0)	0.66
**Race/Ethnicity**				
Caucasian, non-Hispanic	22 (73.3)	16 (80.0)	6 (60.0)	0.02
African American, non-Hispanic	4 (13.3)	0	4 (40.0)	
Asian, non-Hispanic	1 (3.3)	1 (5.0)	0	
Hispanic	3 (10.0)	3 (15.0)	0	
**BMI, kg/m^2^**^a^	23.5 (17.6‒41.1)	23.0 (17.8‒40.6)	23.9 (17.6‒41.1)	0.96
**Scalp involvement (%)**	100 (10‒100)	90 (10‒100)	100 (all)	0.03
**Disease duration**^a^**, yr**	5.0 (0.5‒45)	4.5 (0‒28)	8.5 ( 2‒4 5)	0.14
**Current episode duration**^a^**, yr**	1.0 (0.5–21)	1 (0‒7)	3.5 (1‒21)	<0.001
**Other autoimmune disease**	15 (50.0)	13 (65.0)	2 (20.0)	0.05
Thyroid disease	6 (20.0)			
Inflammatory Bowel Disease	3 (10.0)			
Arthritis	2 (6.7)			
Vitiligo	2 (6.7)			
Psoriasis	1 (3.3)			
Atopic dermatitis	1 (3.3)			
**Neutrophilic dermatosis**	1 (3.3)			
**Nail changes (n = 24)**	6 (20.0)	2 (11.1)	4 (66.7)	0.02
**Previous systemic therapies**				
Systemic steroids	20 (66.7)	13 (65.0)	7 (70.0)	1.00
Methotrexate	6 (20.0)	4 (20.0)	2 (20.0)	1.00
Cyclosporine	2 (6.7)	2 (10.0)	0	0.54
**Treatment duration, months**	18.6 (3.1–68.9)	28.3 (6.1‒68.9)	6.2 (3.1‒29.6)	<0.001

a When patients started tofacitinib therapy. Continuous variables are presented as median (range) and categorical as number (%).

In the present sample, a long duration of the current AA episode, an increased proportion of scalp involvement, and the presence of nail changes and other autoimmune diseases were significantly associated with a poor response. While all Hispanic patients were complete responders, all African American patients were non-complete responders. All 4 patients considered to have anti-TNF-α-triggered AA had a complete response to tofacitinib upon discontinuation of the anti-TNF-α agent.

Twenty patients achieved a complete response to tofacitinib (Table 2). Four had a few episodes of mild to moderate exacerbation that responded either to intralesional steroid injections or an increased dose of tofacitinib (10 mg BID). Ten patients had either no, minimal, or partial response to tofacitinib.

**Table 2:** Response to systemic tofacitinib.

Treatment duration, months	18.6 (3.1–68.9)
Treatment lasting more than 12 months	19 (63.3)
Treatment lasting more than 30 months	10 (33.3)
Response to treatment	
Complete/almost complete	20 (66.7)
Partial	6 (20.0)
Minimal/no response	4 (13.3)
First time response was noted, weeks	12.0 (4.3–61.0)
Full regrowth achieved, weeks	32.9 (19.6–176.1)
Response duration, weeks	77.9 (5.3–273.3)
Need for additional therapy	
Intralesional steroid injection	7 (23.3)
Prednisone taper	2 (6.7)
Increase tofacitinib dosage (10 mg BID)^a^	16 (53.3)
Oral minoxidil (2.5 mg QD)	1 (3.3)

a Initial dose was 5 mg BID, increased to a maximum of 10 mg BID, if regrowth was not achieved at follow up. Continuous variables are presented as median (range) and categorical as number (%).

Most patients received long-term therapy. Nineteen patients completed a year of therapy and 10 patients had been treated for more than 2.5 years. Initial signs of regrowth were noted in 12 (4‒61) weeks.

Seven patients discontinued tofacitinib for 2‒4 weeks. None experienced significant worsening and continued to respond when tofacitinib was restarted. Two other patients, initial responders, after discontinuing tofacitinib due to lack of insurance, promptly lost all regrown hair.

Table 3 displays the safety information for this cohort. Two patients had a severe infection requiring hospital admission, including one patient hospitalized for a UTI who had Crohn’s disease with a fistula and one obese patient with severe COVID. Two patients discontinued because of adverse events: an acute skin rash and progressively increased liver enzymes and lipids. Aside from a basal cell carcinoma diagnosed during the fifth year of tofacitinib, there were no other malignancies reported. There were no reports of thrombosis or tuberculosis. Four patients discontinued tofacitinib because of absent or minimal response.

**Table 3:** Safety – Adverse events noted during dermatology consultations.

AE	Number of patients	Comments
Severe infection	2	Both admitted to the hospital because of complicated UTI and COVID-19 pneumonia, respectively
COVID	3^a^	Only 1 was admitted to the hospital and was later discharged.
HPV infection	3	
Herpes Zoster	1	
Tuberculosis	0	
Increased Lipids	11^b^	1 Discontinued therapy^c^
Increased LFTs	2	1 Discontinued therapy^c^
Acneiform Eruption	3	Mild
Maculopapular Eruption	1	Discontinued
DVT ‒ Thrombosis	0	
New Malignancy	1	1 BCC

a Out of 9 patients tested for COVID-19.

b Including 2 patients with pre-existing hyperlipidemia that became worse after receiving tofacitinib.

c Same patient, therapy had not been effective after 8 months. After an increase in LFTs and lipids, tofacitinib was discontinued.

This retrospective study demonstrated that tofacitinib is an effective treatment option for AA. Long-term continuous therapy produces a stable and durable response. Even for patients that temporarily held therapy, the response was either maintained or restored.

A retrospective analysis of 90 patients demonstrated that, among 65 patients with a current episode duration of 10 years or less, 20% were complete responders (> 90% change in SALT score).^6^ In other studies, a response greater than 50% change in SALT score has been demonstrated in 32%‒56% of patients^2^, ^3^, ^7^, ^8^ and a 90% change in 28% of patients.^7^ The proportion of complete responders in the present study was much higher (67%), possibly related to much longer follow-up time. Some of the patients achieved complete regrowth only after 2 years of therapy.

Among potential factors that may affect treatment response, an increased proportion of scalp involvement, longer duration of current episode, presence of nail changes, or other autoimmune diseases were significantly associated with non-complete response in the present sample. There was also a significant association between race and response, with all African American patients being among non-complete responders and all Hispanic patients being complete responders. In other studies, AA subtype, current episode and disease duration, and age at first episode were associated with efficacy. Nail involvement and the presence of other autoimmune diseases were not evaluated in these studies.^2^, ^7^ Race has never been assessed in prior studies.

Among patients that temporarily discontinued tofacitinib, all had minimal or transient hair loss. Three patients held their therapies for a few weeks, because of COVID-19. None had significant hair loss. Conversely, two patients lost all regrown hair, after treatment ended because of a lack of insurance. In a prospective trial, about a third of study patients were available at a 3-month follow-up after tofacitinib discontinuation. Hair loss was present in all of them after a median of 2 months.^2^

The safety data in the present cohort was promising. Recent retrospective studies analyzing the safety of tofacitinib for other indications support a positive safety profile of tofacitinib longer-term.^9^, ^10^ These retrospective studies, along with the positive safety data, provide us with a better safety outlook in regard to the long-term real-world treatment of AA with tofacitinib.

Limitations of this study include selection bias, variable follow-up times, lack of controls and small sample.

In conclusion, oral tofacitinib may be an effective long-term treatment for AA in carefully selected patients.

## Financial support

None declared.

## Authors’ contributions

Xinaida Taligare V. Lima: Approval of the final version of the manuscript; critical literature review; data collection, analysis and interpretation; manuscript critical review; preparation and writing of the manuscript; statistical analysis; study conception and planning.

Melissa Bambery: Approval of the final version of the manuscript; critical literature review; manuscript critical review; preparation and writing of the manuscript; study conception and planning.

Maria Beatrice Alora: Approval of the final version of the manuscript; critical literature review; effective participation in research orientation; intellectual participation in propaedeutic and/or therapeutic management of studied cases; manuscript critical review; preparation and writing of the manuscript; study conception and planning.

## Conflicts of interest

Xinaida Taligare V. Lima serves on the speakers’ bureau for Abbvie.

Melissa Bambery has no relevant conflicts of interest.

Maria Beatrice Alora has been an investigator for Abbvie, Janssen, Celgene, Eli Lilly, Pfizer, Inc., Novartis, Concert Pharmaceuticals.
